# NOTIFICATION: D. Teng, Y. Xu, Q. Yang, W. Zhang, “The Efficacy and Safety of Laparoscopic Common Bile Duct Exploration With Primary Duct Closure for Cholecystolithiasis Combined With Choledocholithiasis,” *Clinical Case Reports* 12, no. 9 (2024): e9414. https://doi.org/10.1002/ccr3.9414

**DOI:** 10.1002/ccr3.9683

**Published:** 2024-12-07

The above article, published online on September 4, 2024, on Wiley Online Library (wileyonlinelibrary.com), was published as a case study and included a sample size that is greater than the maximum required by the journal. The article was peer reviewed and published, however, it is not a case study as defined by the journal.

